# Light on osteoarthritic joint: from bench to bed

**DOI:** 10.7150/thno.64340

**Published:** 2022-01-01

**Authors:** Yingying Zhou, Junguo Ni, Chunyi Wen, Puxiang Lai

**Affiliations:** 1 Department of Biomedical Engineering, The Hong Kong Polytechnic University, Hong Kong SAR; 2 The Hong Kong Polytechnic University Shenzhen Research Institute, Shenzhen, China; 3 Research Institute of Smart Ageing, The Hong Kong Polytechnic University, HKSAR; 4 Photonics Research Institute, The Hong Kong Polytechnic University, HKSAR

**Keywords:** Osteoarthritis, photoacoustic imaging, photothermal therapy, cartilage, synovium

## Abstract

Osteoarthritis (OA) is one of the rapidly growing disability-associated conditions with population aging worldwide. There is a pressing need for precise diagnosis and timely intervention for OA in the early stage. Current clinical imaging modalities, including pain radiography, magnetic resonance imaging, ultrasound, and optical coherent tomography, are limited to provide structural changes when the damage has been established or advanced. It prompts further endeavors in search of novel functional and molecular imaging, which potentially enables early diagnosis and intervention of OA. A hybrid imaging modality based on photothermal effects, photoacoustic imaging, has drawn wide attention in recent years and has seen a variety of biomedical applications, due to its great performance in yielding high-contrast and high-resolution images from structure to function, from tissue down to molecular levels, from animals to human subjects. Photoacoustic imaging has witnessed gratifying potentials and preliminary effects in OA diagnosis. Regarding the treatment of OA, photothermal-triggered therapy has exhibited its attractions for enhanced therapeutic outcomes. In this narrative review, we will discuss photoacoustic imaging for the diagnosis and monitoring of OA at different stages. Structural, functional, and molecular parameter changes associated with OA joints captured by photoacoustics will be summarized, forming the diagnosis perspective of the review. Photothermal therapy applications related to OA will also be discussed herein. Lastly, relevant clinical applications and its potential solutions to extend photoacoustic imaging to deeper OA situations have been proposed. Although some aspects may not be covered, this mini review provides a better understanding of the diagnosis and treatment of OA with exciting innovations based on tissue photothermal effects. It may also inspire more explorations in the field towards earlier and better theranostics of OA.

## Introduction

Being one of the rapidly growing disability-associated conditions worldwide, Osteoarthritis (OA) is a leading cause of chronic pain and poor quality of life especially for older adults 1. Currently, there is no effective cure for OA when articular cartilage damage is established 2, which prompts an urgent need for early detection and intervention of the disease prior to irreversible joint damage.

The key problem in early detection and intervention of OA is a historical observation on the disparity of structural damage and symptomatic severity 3. Current imaging modalities, radiography (X-ray), positron emission tomography (PET), magnetic resonance imaging (MRI), optical coherence tomography (OCT), and ultrasound (US) 4, 5, are limited to provide structural information and fail to elucidate functional deficit and pain severity. The capacities of these imaging modalities are summarized in Table 1. Among them, X-ray is a classic method to assess bony changes, especially joint space width (JSW) and osteophytes, but it is radiative and has limitations in imaging soft tissue and subchondral structures 6, typical symptoms of early OA. For MRI, US, and OCT, they are nonradioactive and provide more detailed imaging information that is impossible with X-ray, enhancing the diagnosis of OA. However, MRI is costly and its accessibility is limited, having a long waiting list for public hospital patients even in some developed regions like Hong Kong. Moreover, the imaging modality is contraindicated for patients wearing implanted devices 6. For US imaging, it has low contrast, strong boundary effects, and insufficient sensitivity to early OA changes, being lack of standardized deifications 6. OCT, in comparison, achieves a better balance among cost, sensitivity, and contrast, but suffers a shallow imaging depth due to strong scattering of light in tissue, making it difficult for noninvasive diagnosis of OA at depths. Although the PET can obtain active metabolism in articular and periarticular tissues, thus allowing evolution of metabolic changes within the bone. This method lacks the needed spatial resolution for studying the microenvironment and relies on radioactively labeled exogenous tracers.

**Figure 1:**
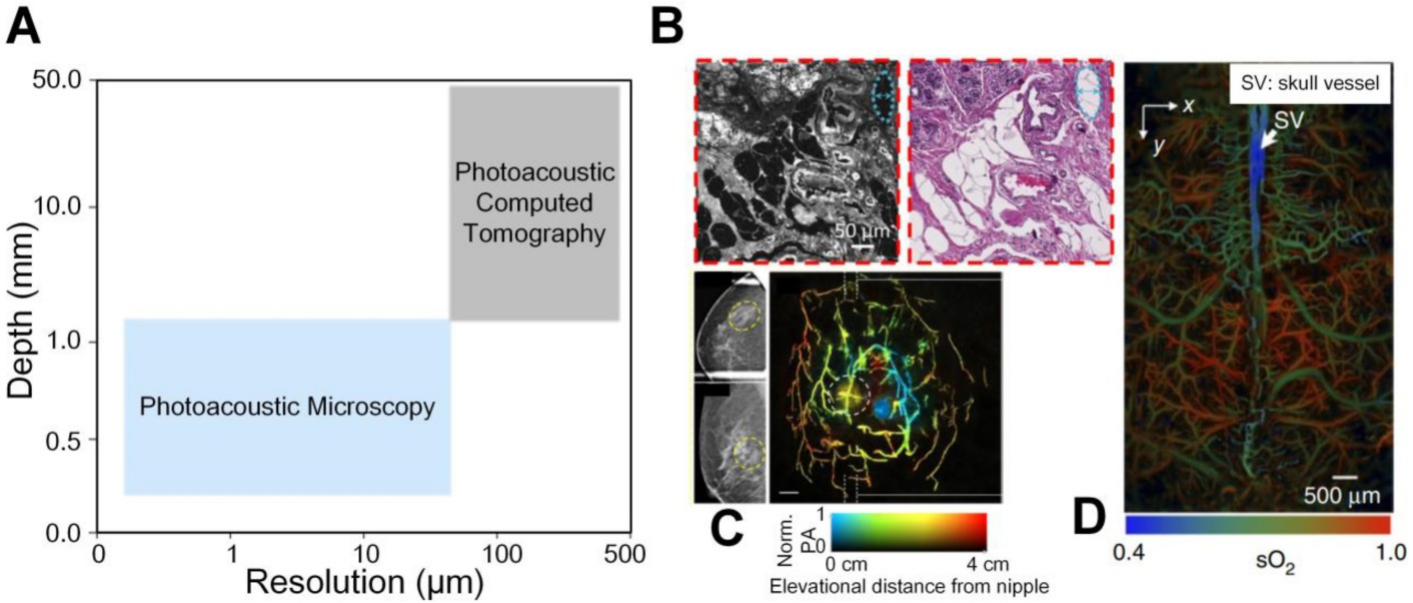
(A) A continuum from micro to macro by photoacoustic imaging. (B) An example of micro-scale structural photoacoustic imaging. Photoacoustic microscopy and hematoxylin and eosin-stained figures of normal human breast tissue 17. (C) An example of macro-scale structural photoacoustic imaging. Photoacoustic computed tomography of human breast cancer 18. (D) An example of micro-scale functional photoacoustic imaging. Oxygen saturation of mouse brain 19. Adapted with permission from 17-19. Copyright © 2017, Copyright © 2018, and Copyright © 2015 Springer Nature.

The aforementioned imaging modalities including X-ray and MRI have achieved excellent penetration for anatomical imaging from macro-scale aspect. In addition, the PET and single-photon emission computed tomography (SPECT) are highly sensitive to the radiolabelled molecular probes, which realizes deep penetration. The optical microscopes can describe biological phenomena with subcellular and subcellular resolution in detail at superficial depths from micro-scale side. However, different imaging contrast mechanisms and length scales of these imaging tools hinder the multi-scale study of biological problems. Therefore, a continuum from micro to macro imaging is desired to be established in life science. Photoacoustic (PA) imaging is a hybrid imaging modality based on photothermal effects. Through converting diffused light into non- or weak-scattered ultrasonic waves, PA imaging combines the advantages of optical contrast and ultrasound detection 7-10. It can yield high-contrast and high-resolution images of different subsurface tissues to probe molecular events or changes, and it has been proven to be capable of multiscale imaging from micro to macro fields in life science, sharing a consistent contrast mechanism 11. Depending on how photoacoustic signals are received and reconstructed to form the image, photoacoustic imaging can roughly be divided into two categories, photoacoustic microscopy (PAM) and photoacoustic computed tomography (PACT). As illustrated in Figure 1(A), usually PAM has a higher resolution but a shallower penetration depth compared with PACT due to trade-off between the optical spatial resolution and penetration depth. Combining PAM and PACT, photoacoustic imaging can achieve a multiscale ability, as shown in Figures 1(B-D). More importantly, many endogenous tissue constituents, such as oxyhemoglobin, deoxyhemoglobin, and water, possess distinctive optical absorption spectra and hence can be used as natural contrasts to be captured under optical illumination of different wavelengths to indicate status of targets, providing label-free functional and molecular specific information 12. In scenarios where the endogenous contrasts cannot yield sufficient signal-to-noise ratio (SNR), for example to detect cancer from blood rich organs like liver, exogenous contrast agents can be added and accumulate at the region of interest, which leads to increased contrast and highlighted object imaging 13-16.

Benefiting from its attractive advantages, photoacoustic imaging has been widely used in early diagnosis and treatment monitoring of a variety of diseases, such as cancers 18, 20-22, vascular diseases 23, 24, and brain conditions 25, 26. Lin *et al.* developed a single-breath-hold PACT system and successfully revealed a higher blood vessel density associated with tumors in human breasts with high resolution in deep penetration depth 18. Jo *et al.* reported an *in vivo* quantitative tumor pH mapping nanotechnology with nanoprobes by multi-wavelength PA imaging, which holds great potential for both research on and clinical management of a variety of cancers 27. Liu *et al.* successfully delineated *in vivo* brain gliomas with the help of exogenous contrast through near-infrared PA imaging 26. Long before the structural and functional parameters including vascular morphology, blood oxygenation, blood flow and oxygen metabolism have been realized by PA imaging 19. Recently, there also has a growing body of interests in application of PA imaging for OA research in both animals and humans. Hereby, we discussed current development of PA imaging at the crossroad of optics, and biomaterials and biomedicine, and how the PA imaging applied in the early stage of OA for diagnostic purpose.

Precise diagnosis of early-stage OA also benefits timely treatment of the disease. Current state-of-the-art photothermal technology has emerged as a novel OA therapy 28, 29. The mechanism of photothermal therapy (PTT) in some way is akin to PA, i.e., converting light to heat due to tissue absorption. Thus, PTT can achieve non-invasive treatment, which has already been verified in many studies 30-32.

In this narrative review, we aim to discuss the mechanism and feasibility of noninvasive photoacoustic diagnosis of OA in its early stage and monitoring the disease progression, including both structural and microenvironment alterations. The application of photothermal-triggered drug release for OA is also discussed herein. Lastly, relevant clinical applications are discussed. It is hoped that the review, although some aspects may not be covered due to the limit of manuscript space, can deepen the understanding of diagnosis and treatment of OA with photothermal effects and inspire more explorations in the field towards more clinical trials.

## Evolution of osteoarthritis

**Figure 2:**
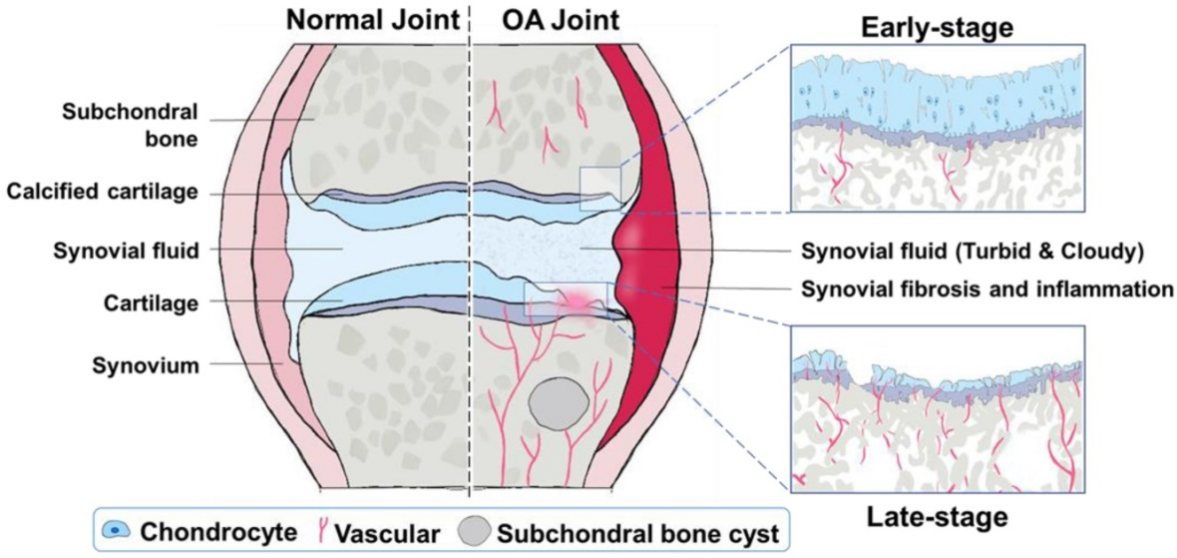
Illustration of structural and microenvironment transformations during OA progression. Compared with normal joints, OA joints will undergo different degrees of changes with the OA progression. In early-stage OA, the surface contour of cartilage is lightly unregulated, the superficial zone of articular cartilage splits slightly, the subchondral bone plate is thinner yet more porous, and a few vessels erode into the avascular cartilage. In late-stage OA, the whole cartilage structure is completely destroyed, which is usually accompanied with subchondral bone sclerosis, joint space narrowing, osteophyte formation, and more vessels erosion into the cartilage.

OA is one of the most common chronic arthritic diseases, and it is also the main cause of disability and joint dysfunction in the elderly nowadays 33. The pathophysiology of OA is very complicated. It is related to age, gender, obesity, diet, genetic factors, and many other factors at the individual level 34-36. The typical characterization of OA is the degeneration of articular cartilage and elevated chondrocyte mortality 37. Possible reasons include mechanical and biological factors that lead to degradation and synthesis imbalance of chondrocytes, extracellular matrix, and subchondral bone 38, 39. These structural and functional variations are inherently accompanied with optical properties changes at single or multiple wavelengths, which can potentially be sensed or mapped by PA imaging. Therefore, this section discusses the evolution of joint OA from structure damages and microenvironment changes, respectively (Figure 2), forming the physiological basis of PA diagnosis of OA joints.

### Structure damages of joint tissue in OA

Our human body is composed of many joints, whose structures are usually complex and distinct from one another. Among these joints, many, such as knee, finger, hip, and spine, could develop OA symptoms 40-42. These symptoms, according to extensive etiological and pathological studies in the field, often involve structure change or damages in the joints, which gradually evolves and emerges as the whole joint disease 39, 43. For instance, knee joints are mainly organized by articular cartilage, bones (femur, tibia and fibula), synovium, tendons, ligaments, and meniscus 44. Abnormalities of these constituents are often associated with OA progression 43. Hence, one of the key characteristics of OA diagnosis is whether these structural changes can be imaged or sensed, for which PA could play a promising role.

Especially, articular cartilage is a critical pivotal part of joints; its damage is observed in nearly all OA patients, and hence its degeneration is often referred to as one of the hallmarks of OA 45. Macroscopically, articular cartilage changes associated with OA include, but are not limited to, cartilage softening, fibrillation, and erosion 46. Although the morphology changes are not significant at the early stage of OA, histologically, the superficial zone of articular cartilage appears to change first, which can be visually observed 47. This damage will become more serious as the OA condition worsens, from surface contour lightly unregulated until the whole cartilage structure completely destroyed 48. Likewise, observing the morphology of subchondral bone altered is another essential index in OA diagnosis. In past decades, large efforts have been made to demonstrate the importance of abnormal subchondral bone remodeling in OA development 49, 50. It has been reported that subchondral bone integrity will be influenced with the OA development and subchondral bone plate thinner, and more porous could be detected in early OA 51. Moreover, studies no matter in animal models or in specimens from OA patients emphasize sclerosis of subchondral bone, joint space narrowing, and osteophyte formation in late stages of OA 52-54. Concomitantly, swelling of synovium and the injury of other components (i.e., meniscus, ligaments, etc.) of joint are also the major sites of macroscopic changes in the OA process 43, 55, 56. As such, PA could be used to diagnose and evaluate the severity of OA by imaging the structure of joints and its changes.

### Functional (microenvironmental) changes in OA joints

That said, OA is a slow progression disease; the deformation of anatomical structures in the joints is slight and might be hard to be detected at the initial stage 57. In comparison, microenvironmental communications happened throughout the entire OA progression, presenting parameter variations before the morphology injuries 58. These microenvironment changes could lead to alterations of tissue optical properties, such as absorption and/or scattering spectrum, which provides potentials for early diagnosis of OA joints with photoacoustic methods.

Abundant extracellular matrix and chondrocytes embedded within it compose articular cartilage 59. For natural and healthy cartilage, water, collagen Ⅱ, and proteoglycan are the primary compositions 46. At the initial stage of OA, the form of proteoglycans begins to change, causing shorter chain length and lower percentage of normal macromolecular complexes 46. Meanwhile, the water content increases in the cartilage matrix, which softens the articular cartilage and changes the biomechanical feature 60. These changes further induce chondrocyte abnormal activities, oversecrete proteolytic enzymes and inflammatory cytokines, and finally disrupt the collagen network, such as matrix metalloproteinases (MMPs), nitric oxide (NO), reactive oxygen species (ROS), tumor necrosis factor-α (TNF-α), interleukin-1β (IL-1β), etc 37, 61-63.

In the subchondral bone, the disorder of bone metabolism is the direct consequence of abnormal cell-to-cell communications 64. It is well known that the amount of blood vessels increases in subchondral bone and vessel erosion into the avascular cartilage with OA progression 65, 66, as illustrated in Figure 2. A rabbit's study demonstrates that angiogenic activity of subchondral bone achieves the highest level in the early to the progressive stage OA, and then decreases back to the normal level in the late stage of OA 67. Subsequently, more recent research finds that subchondral bone angiogenesis is induced by abnormal rise of platelet-derived growth factor-BB 68. Moreover, accumulating evidence has shown that hypervascularization and subchondral bone edema occur earlier than cartilage lesions 69-72, influencing the microenvironment like water content, hemoglobin concentration, and blood oxygenation, which could be used as the early diagnostic indicator of OA.

*Taken together*, the structure alterations due to OA are mainly reflected by volume loss of articular cartilage, thickening of subchondral bone, and the formation of osteophyte, whose PA imaging features could be used as the criteria for the diagnosis of OA. However, these visual changes of joints usually do not emerge until the late stage of OA, which limits the treatment choice of patients. Detecting initial OA-like variations in joints as early as possible is highly desired yet still challenging in clinical diagnosis. Fortunately, in addition to structure imaging, PA is also capable to track the changes of microenvironment with disease progression based on the expression level of molecules 73-75, which strongly supports the hypothesis of PA for early diagnosis of OA as well as dynamic monitoring of OA progression.

## Photoacoustic diagnosis of osteoarthritis

### Structure changes in OA observed with PA

As different tissue constituents as well as their alignment and distribution result in their characteristic absorption spectra, PA imaging can be applied to observe the structural changes associated with OA based on their wavelength-dependent property 76.

#### Sponge bone

Izumi *et al*. designed an ultrasonic and photoacoustic dual-mode imaging platform to investigate the divergences between normal rat knee joints and OA joints 77, 78. In their findings, compared with normal joints, the sponge layer expresses considerably stronger PA signals in the OA joints, while the US reflections are almost identical in two scenarios. The trend can be seen more clearly from the quantitative averaged US and PA intensities in the sponge layer between the healthy joints and OA joints: the PA signal amplitudes induce obvious differences between normal and OA joints, while the US values show much fewer variations. The enhanced contrast in PA is probably associated with the increased optical absorption from more blood due to vascularization and inflammation as illustrated in Figure 2. It also suggests that PA potentially provides a better indicator than US does to visualize the sponge bone of OA from normal joints.

#### Cartilage and subchondral bone

**Figure 3:**
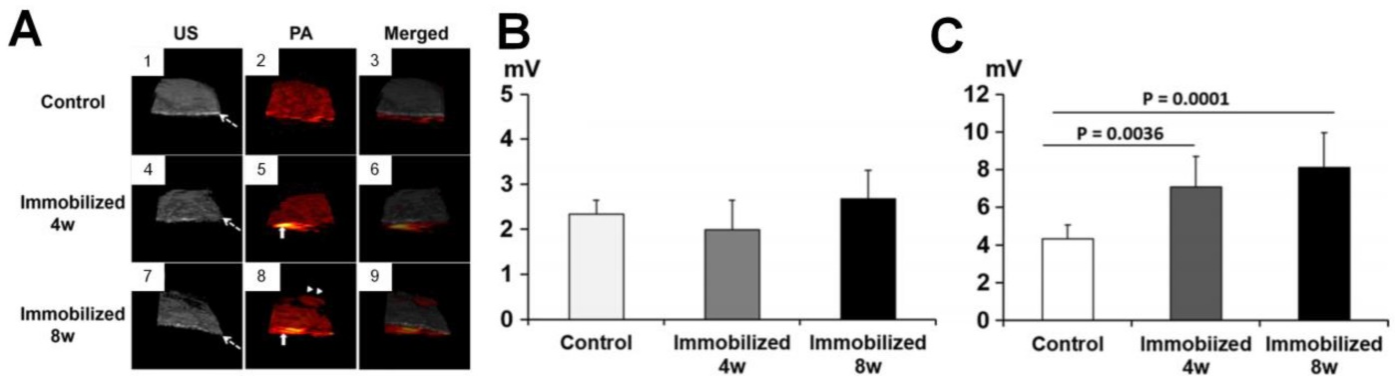
(A) US, PA and the merged images of knee joints in different stages. (B-C) Quantified PA amplitudes at different stages from cartilage and subchondral bone, respectively. Figures adapted with permission from Ref 79. Copyright © 2015, Elsevier.

In addition to the elevation of PA signals in the sponge bone of OA joints compared to normal joints, Hagiwara *et al*. investigated the PA/US signals changes in the subchondral bone and cartilages by sequential monitoring of immobilized knees in rats 79. The white dashed arrow in Figure 3(A) points to the subchondral bone and the upper part of it is cartilage. The US mode in the PA system demonstrates that the surface of the articular cartilage becomes irregular with a longer duration of immobilization, while the PA mode shows a dramatic signal increase in the subchondral bone with long immobilization. Quantified PA changes are shown in Figures 3(B-C). As seen, the PA signals show limited differences in cartilages before and after immobilization. In contrast, significant variations are observed in the subchondral bone, which indicates that the PA signal intensities elevates in the subchondral bone with the time of immobilization to the keen joints. Therefore, the combined US/PA information can be utilized to assess the OA joints from the cartilage and subchondral bone changes.

### Functional (microenvironmental) changes in OA obtained with PA

The early diagnosis of OA can help patients relieve pain timely and improve therapy outcomes. However, early-stage OA may exhibit few or very light changes in structures, which may not be easily sensed or detected by PA. Under this circumstance, capturing the changes of microenvironmental parameters, such as metabolism, blood information, and optical absorption, in OA joints possesses critical significance.

#### Vascularity and hypoxic status

**Figure 4:**
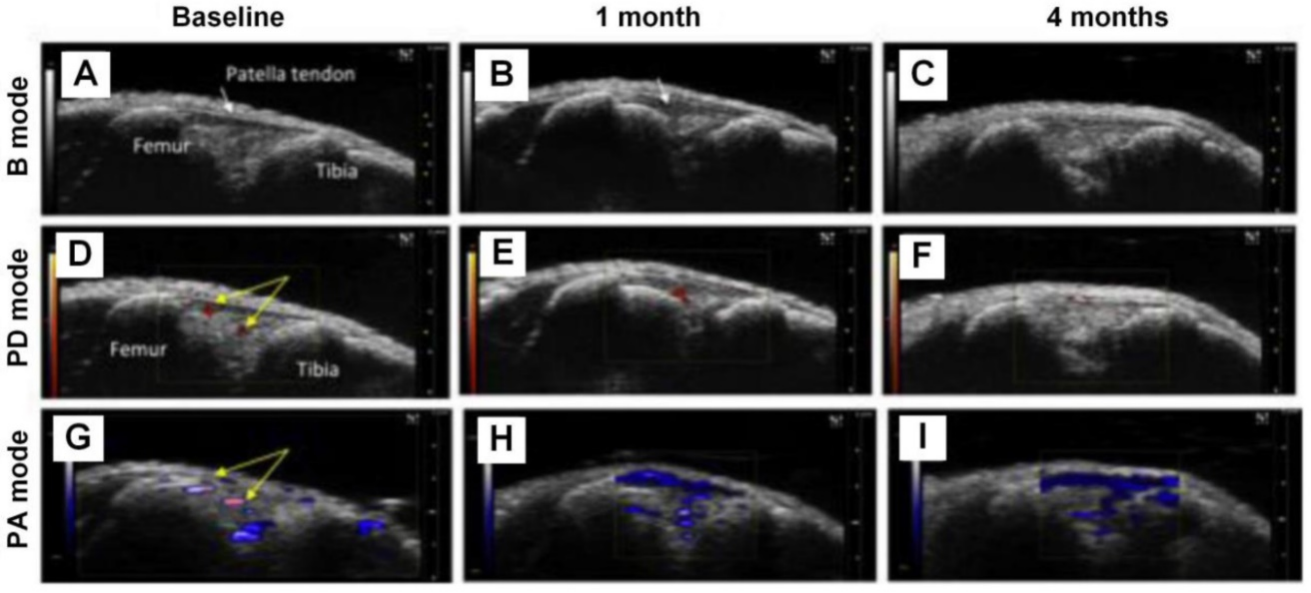
DMM knee joints at different stages being visualized with B mode ultrasound, PD mode ultrasound, and PA imaging, respectively. (A)-(C), B mode ultrasound images of the knee joints at baseline, 1 month, and 4 months, respectively. (D)-(F), PD mode ultrasound images of the knee joints at baseline, 1 month, and 4 months, respectively. (G)-(I), PA images of the knee joints at baseline, 1 month, and 4 months, respectively. Figures adapted with permission from Ref 80. Copyright © 2018, Elsevier.

Inflammation of the synovium is another symptom of OA. Liu *et al*. proposed to use PA imaging, combined with B mode ultrasound and Power Doppler (PD) ultrasound, to monitor the synovial tissue vascularity and hypoxic status changes in the knee joints 80. The results are shown in Figure 4, from which one can see that in 1 month and 4 months post destabilization of medial meniscus (DMM) surgery, PA signals increase significantly. Moreover, based on PA signals obtained at two optical wavelengths (750 nm and 850 nm in this experiment), the hemoglobin oxygen saturation information can be extracted, which suggests a dramatic decrease in the DMM knee joints from normal joints, and the degree of declination is closed related with the severity of OA (lower oxygen saturation in severer OA joints). These results are well verified in the B mode and PD mode images, demonstrating the feasibility of utilizing PA imaging to monitor the variations of vasculature and oxygen saturation in the OA joints correctly.

#### Hemoglobin concentration, water contents, and acoustic velocities

**Figure 5:**
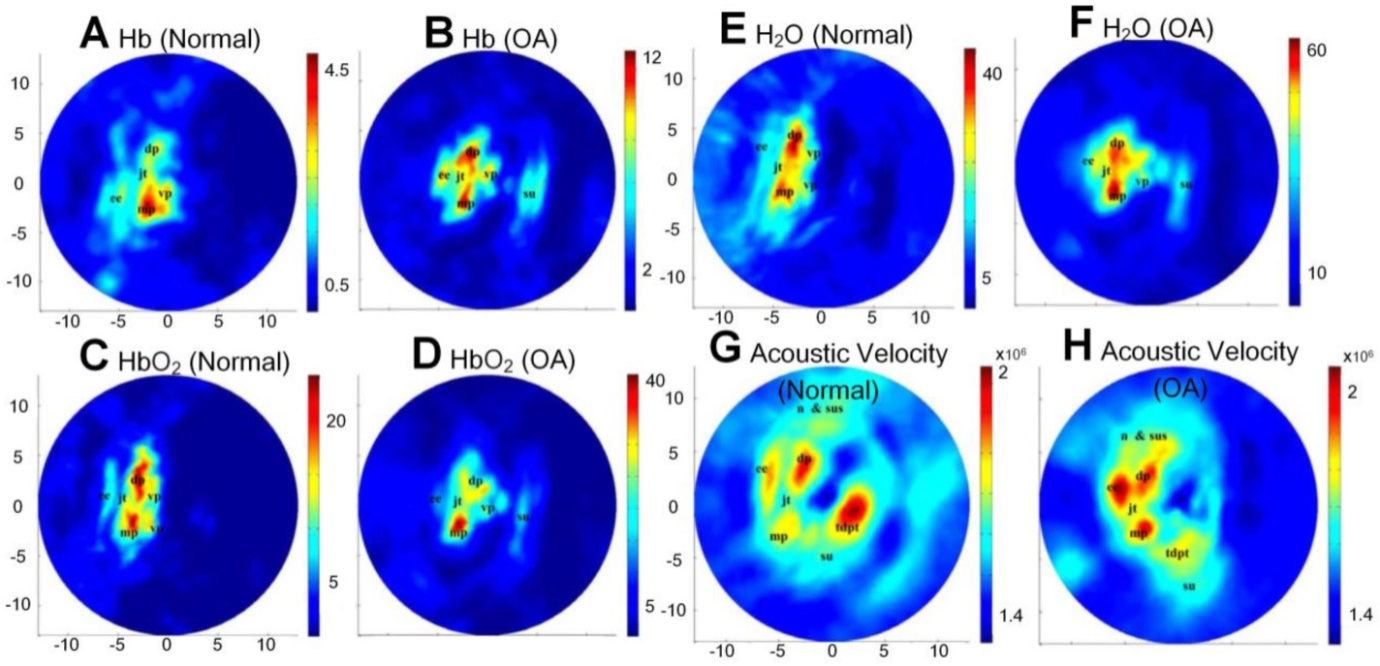
(A)-(H) Different functional parameters (Hb, HbO2, water contents, and acoustic velocity) of normal and OA joints, respectively, through multi-wavelength PA imaging. Figures adapted with permission from Ref. 81 . Copyright © 2010, The Optical Society.

With multi-wavelength PA imaging, Xiao *et al.* quantitatively compared several functional parameters, including oxyhemoglobin (HbO_2_), deoxyhemoglobin (Hb), water contents, and acoustic velocities between normal distal interphalangeal (DIP) and OA joints 81. The extracted parameter images are listed in Figures 5(A-H). As shown, significantly increased water contents, declined oxygen saturation, and elevated acoustic velocity are observed in OA joints as a comparison with normal joints. Later, the same group further modified their system into a 3D multispectral quantitative version and reported the difference of hemoglobin concentration and oxygen saturation in osteoarthritic phalanges and soft joint tissues in joint cavities between the OA and healthy groups 82. An elevated hemoglobin concentrations and declined oxygen saturations in these two different joint tissues were observed, which is consistent with their previous findings 81, 83.

#### H_2_O_2_ expression

**Figure 6:**
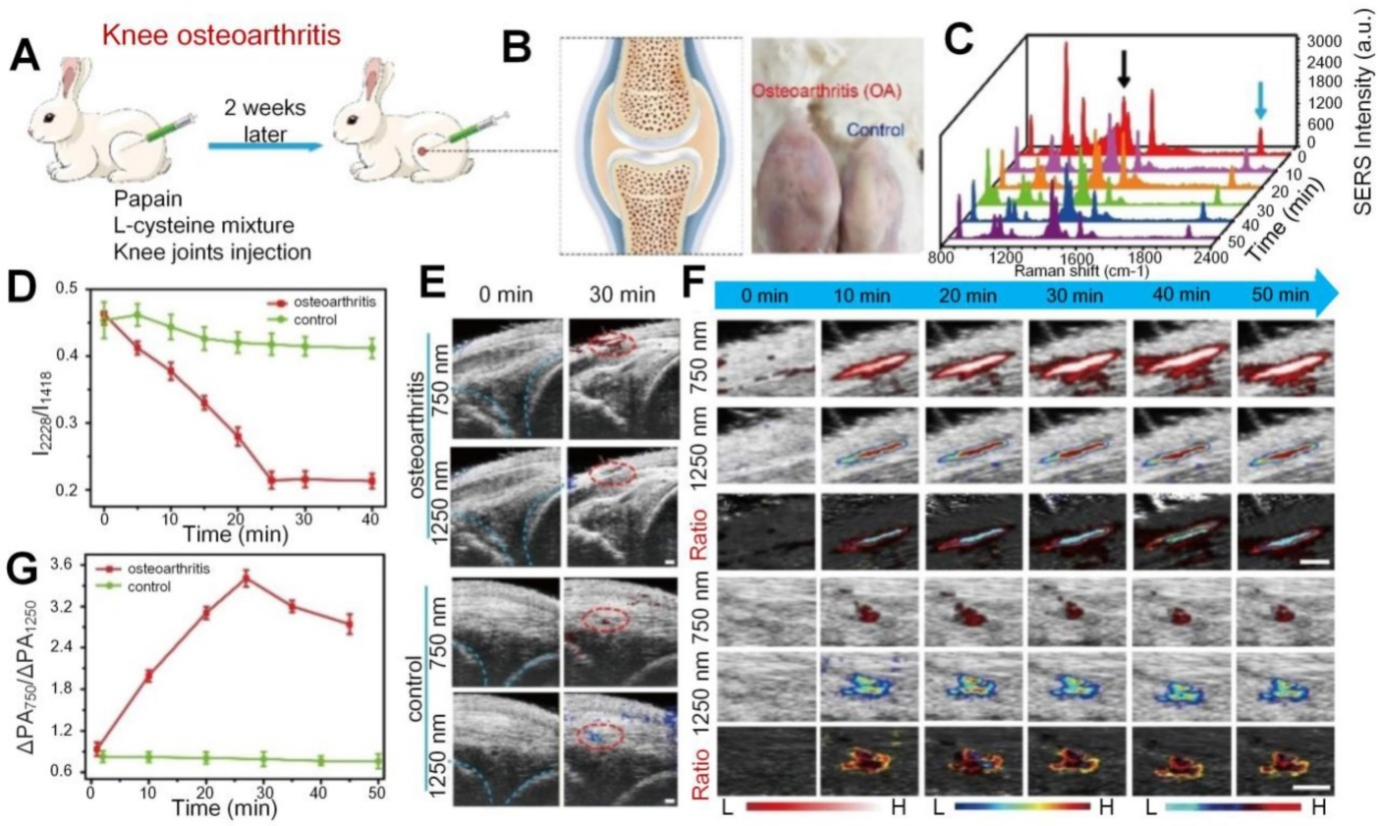
(A) *In vivo* knee OA model establishment process. (B) The cartoon figure (left) of knee OA and the photos (right) of OA and normal knees. (C) SERS spectra. (D) I2228/I1418 SERS intensity change versus time. (E) Ultrasound (US)/PA images of the OA and normal knees. PA signals are marked in red circles. (F) 750 nm, 1250 nm, and the ratiometric PA images. (G) Ratiometric PA750/PA1250 value versus time for OA and normal knees. Scale bar is 1 mm in (F). Figures adapted with permission from Ref. 84. Copyright © 2020, Wiley-VCH GmbH.

Apart from the vascular parameters and oxygen saturation changes in the synovial tissue for OA joints, there is typical excessive production of oxidative species, such as H_2_O_2_, at the inflammation sites in the synovium. Based on that mechanism, Li *et al.* proposed a dual ratiometric surface-enhanced Raman scattering (SERS) and PA imaging to monitor and quantify the level of H_2_O_2_ in the knee OA of rabbits with core-satellite nanoprobes 84. As shown in Figure 6, the H_2_O_2_ concentration is much higher in the OA knee, but the I2228/I1418 (the SERS intensity at 2228 nm divided by the SERS intensity at 1418 nm) undergoes significant declination with time after nanoprobes injection into the OA knee while the ratio keeps fairly stable in the normal (control) knee. For PA signals in the OA knee, it experiences fuzzy changes with time at both 750 and 1250 nm, but the signal ratio (PA_Δ750_/ PA_Δ1250_) between these two wavelengths does show an obvious growth after injection, which also suggests increased H_2_O_2_ level. The study also suggests the combination of SERS and PA imaging as well as their signal ratios help to precisely locate the OA position. Later on, a similar study was realized by the same group with only PA imaging enhanced by nanoprobes 85. In this study, the H_2_O_2_ expression at different stages of OA is quantified based on the ratios of PA signals at two wavelengths. The results suggest that in the OA joints, the signal ratio is significantly different from the normal joints due to higher expression of H_2_O_2_ and the absorption shifting of nanoprobes.

#### Viscoelastic property

**Figure 7:**
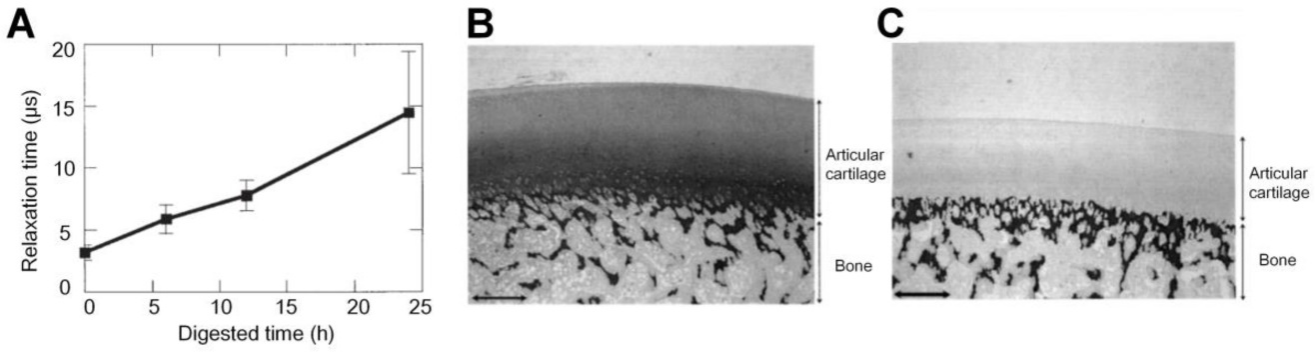
(A) Relaxation time of PA signals at different degrees of cartilage damage. (B)-(C) Histological results of normal porcine cartilage and 12 hours trypsin treated cartilage, respectively. Figures adapted with permission Ref.^86^. Copyright © 2006, Wiley-Liss, Inc.

In addition to the aforementioned functional parameters, PA can also be applied to assess the viscoelastic properties of cartilage damaged at different degrees. Ishihara *et al*. developed a PA viscoelastic tissue measurement method to diagnose cartilages in the OA joints 86 and to evaluate the viscoelastic property of the tissue-engineered cartilages 87-90. As shown in Figure 7, the relaxation time of PA signals, which can be used to compute the viscoelastic characteristics of tissue, is approximately a linear function of the digested time of trypsin treatment. The trypsin was used here to simulate the damage degree of cartilages, which was confirmed with the histological results shown in Figures 7(B-C). These results demonstrate the PA viscoelastic measurement method can be used to delineate the degree of cartilage damage in OA. Ukai *et al*. also found that the laser-induced PA (LIPA) measured viscoelastic results had an approximately (positive) linear relationship with the cartilage damage grade, which is well consistent with the MRI results discussed in the same study 91.

### Probe-enhanced PA imaging of OA

**Figure 8:**
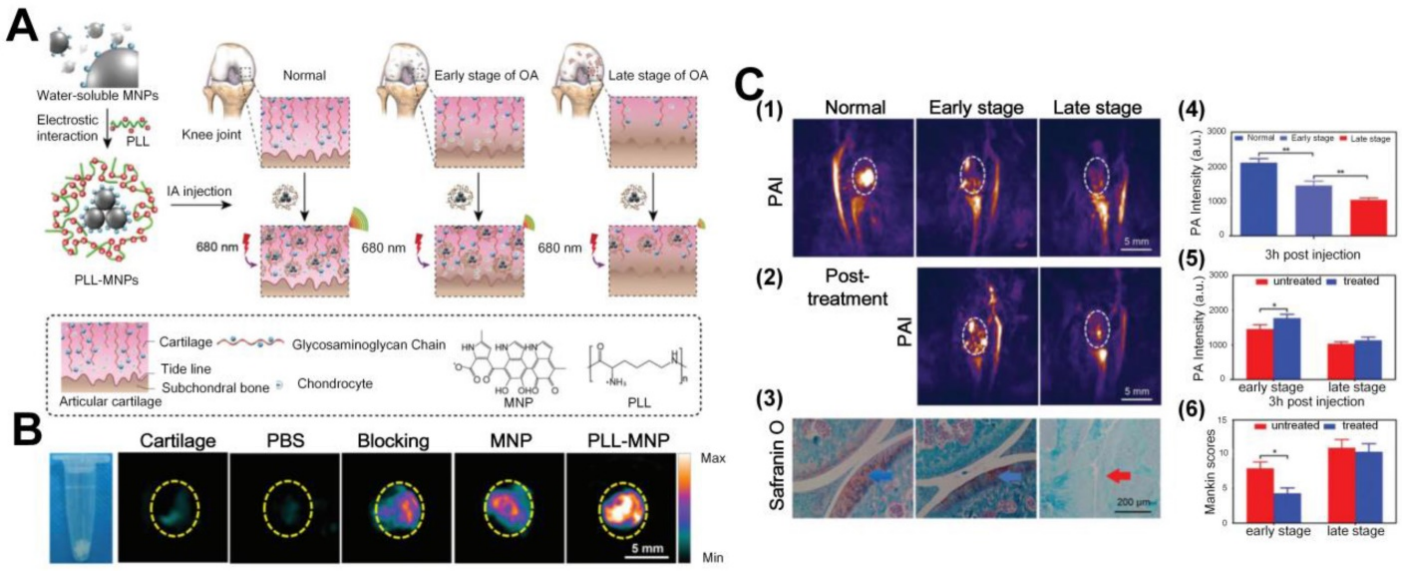
(A) Schematic diagram of the preparation of PLL-MNPs and the mechanism of diagnosis of OA cartilage degeneration with PAI. (B) PA images of cartilage after immersion in different contrast media for 24 hours. Scale bar: 5 mm. (C) PA images of early and late stages of OA with PLL-MNPs, PA post-treatment monitoring after hyaluronic acid (HA) injection, and its comparison with Mankin scoring system results. Figures adapted with permission from Ref. 92. Copyright © 2018, Royal Society of Chemistry.

While endogenous tissue constituents, such as cartilage, subchondral bone, water, and hemoglobin, provide inherent potentials for structure or function-based diagnosis of OA joints with PA imaging, extraneous agents, such as nanoparticles, can be applied to achieve enhanced detection contrast, sensitivity, and specificity of OA symptoms. Current photoacoustic nanoparticles applied in OA can be divided into two categories, inorganic and organic. Most of them are multiple modified for specific applications. Inorganic photoacoustic nanoprobes, such as Ag shell coated Pd-tipped gold nanorods (Au-Pd@Ag NR) developed by Ye *et al*. can quantitatively evaluate the inflammation status by H_2_O_2_ levels and precisely treat it with Ag ions release in the OA sites 85. Another probe, core-satellite gold nanostructure, was reported by the same group also can precisely detect the H_2_O_2_ levels in the inflammation positions 84. The reported organic nanoparticles include articular cartilage-target poly-_L_-lysine-encapsulated melanin nanoparticles (PLLMNPs) 92, 93 and inflammatory-bonded PEGylated, phenylboronic acid modified L-DOPA pro-antioxidant (pPAD) 94. Both of them serve as a good photoacoustic contrast to reveal the OA status from different aspects. Take cartilage degeneration as an example; the anionic glycosaminoglycan (GAGs) contents will decline with the severe degree of OA. In 2018, Chen *et al*. 92 designed cationic poly-_L_-lysine-encapsulated melanin nanoparticles (PLL-MNPs) to function as photoacoustic contrast agent that can target the GAGs contents of articular degeneration in OA due to the strong electrostatic interaction between the PLL-MNPs and GAGs. The principle of the probe design is shown in Figure 8(A). The PA signals of PLL-MNPs targeted with GAGs are much higher than normal melanin nanoparticles (MNP) and other control groups, such as PBS injection and blocking at wavelength of 680 nm, as illustrated in Figure 8(B). The *in vivo* qualitative and quantitative results in Figures 8 (C1 and C4) further show that PLL-MNPs in normal joints with high contents of GAGs exhibit PA signals that are about two folds stronger than the signals from the OA joint possessing lower GAGs contents. As shown in Figures 8 (C2 and C5), researchers have also applied this PLL-MNPs for monitoring the treatment outcomes after 4 weeks hyaluronic acid (HA, one kind of drug to improve cartilage degeneration) injection. Note that the nanoparticle-enhanced PA signals become considerably stronger after the treatment for early-stage OA, but for late-stage OA the PA signals experience much fewer variations, indicating better therapeutic efficacy to early-stage OA than to late-stage OA. This result is consistent with the Mankin score study that uses safranin-O to stain GAGs; in this study, a lower Mankin score represents an improved condition. Based on this probe, the same group further tested the ability of PLL-MNPs enhanced PA to differentiate different GAGs concentrations in cartilage explants. It is confirmed that molecularly enhanced PA signals are capable of reflecting the course of OA *in vivo*, which has great significance in the monitoring of clinical therapy 93.

## Photothermal-triggered drug release therapy of OA

**Figure 9:**
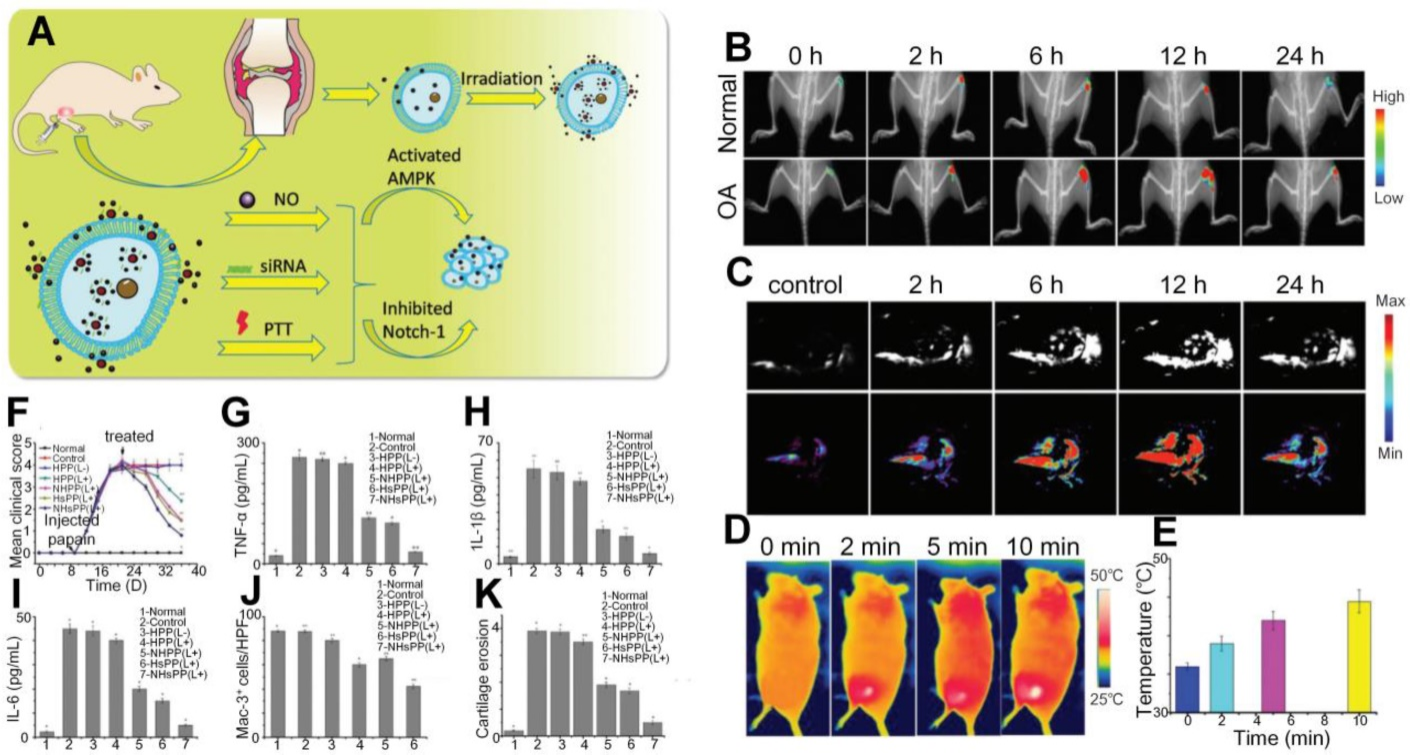
(A) Reaction process of the NHsPP nanoparticles in the OA after light illumination. (B) Spatial distributions of NHsPP nanoparticles in the OA and normal joints at different times. (C) PA signals of the nanoparticles in the OA joints at different time. (D) Nanoparticle-enhanced photothermal images under laser irradiation. (E) The temperatures changes with time under the photothermal effect. (F-K) Semi-quantitative comparisons between different treatment methods as a function of time. Figures adapted with permission from Ref. 29. Copyright © 2019, Royal Society of Chemistry.

Thus far, we have discussed the feasibility of utilizing PA methods to diagnose and monitor the therapeutic outcome of OA. It is hence necessary to introduce photothermal therapy, a treatment method based on photothermal effects and has gained great attention to treat abnormal tissues like tumors 28. The mechanism of PTT in some way is akin to PA, *i.e.,* through converting light to heat due to tissue absorption. That said, PTT typically uses near-infrared light to treat the target with enhanced heat and penetration in tissue, and it must be assured that the surrounding cells and tissues are minimally affected by light illumination. Thus, the usage of exogenous PA contrast agents is usually desired to ensure that the target, accumulated (actively or passively) with molecular probes, has an absorption spectrum that is distinct from that of normal tissues. With light illumination at the absorption peak wavelength of the probe, at the target position, the PTT efficiency can be greatly increased and the treatment outcomes can be ensured, while tissue damage to the surroundings can be suppressed. In current OA treatment, photothermal effect was served as an efficient tool through converting light to heat with the help of multifunctional nanoparticles, which is then activated by the heat to release the therapeutic drugs in OA joints. The multifunctional nanoprobes can transfer the light to heat with high efficiency and carry the therapeutic drug simultaneously. For example, Chen *et al*. synthesized photothermal-triggered NO nanogenerators, NO-Hb@siRNA@PLGA-PEG (NHsPP) nanoparticles, which enabled precise NO, siRNA, and PTT treatment on the inflamed OA simultaneously. The joint treatment significantly enhanced the therapeutic efficiency by suppressing macrophage inflammation in comparison to any individual method alone under 650 nm laser irradiation 29. The reaction process is illustrated in Figure 9 (A), where two signal pathways including up-regulation of AMPK and down-regulation of Notch-1 were triggered under the 650 nm laser irradiation after injection of the NHsPP nanoparticles. Both pathways can promote the macrophage inflammatory response and thus accelerate the anti-inflammatory outcomes. As shown in Figures 9(B-C), in contrast to normal joints, the NHsPP nanoparticles could precisely accumulate at the inflamed sites due to the specific combination of Notch 1-si RNA within a short time and remained in the region for nearly 24 hours. The PTT high efficiency was also confirmed in the OA joints under laser irradiation, as indicated in (D) and (E). Different parameters in OA joints, including mean clinical scores, inflammatory cytokines (TNF-α, IL-1β and IL-6), Mac-3 (a macrophage marker), and cartilage erosion degrees, were compared among different treatment groups, and the results are shown in Figures 9(F-K). All these parameters demonstrate that the NHsPP nanoparticle-assisted photothermic guided effect yield promising therapeutic efficiency and the recovery outcomes are near to the normal groups.

**Figure 10:**
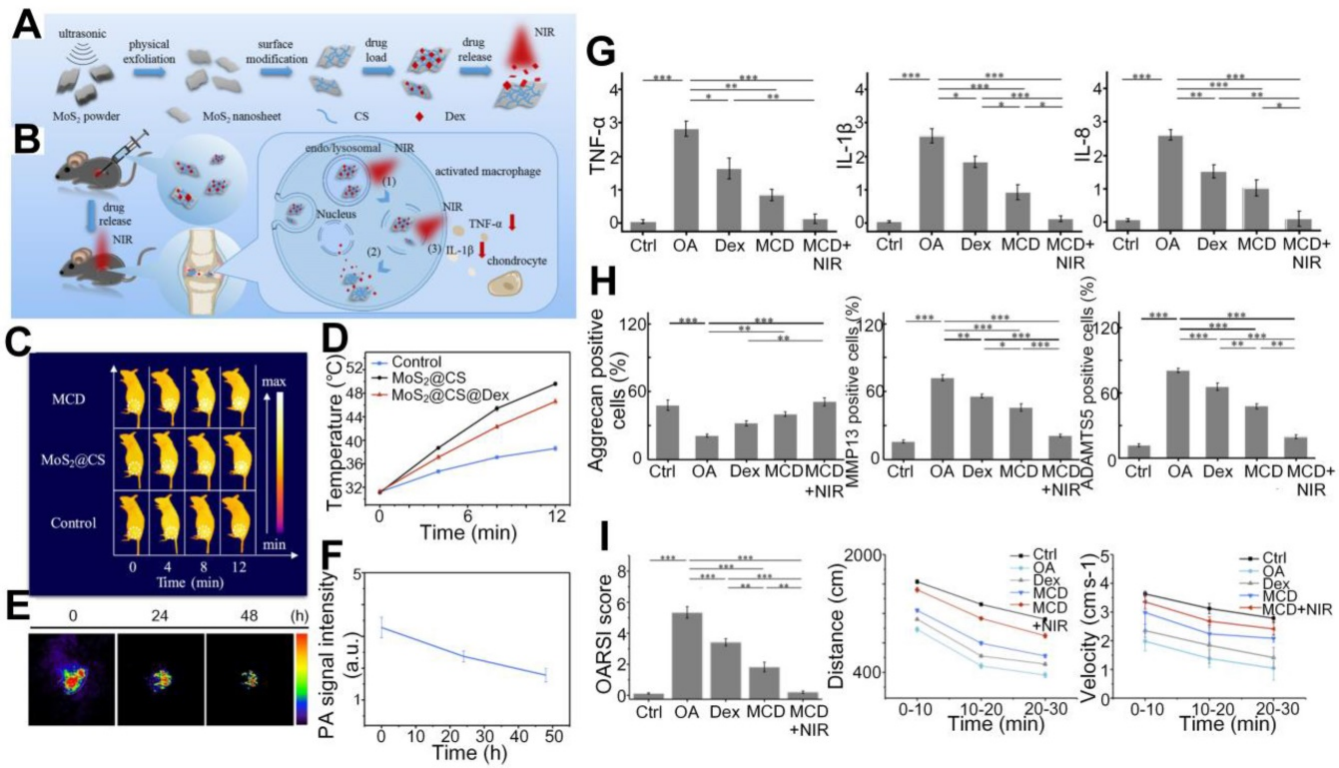
(A) - (B) Schematic diagram of the synthetization and reaction process of the MCD nanoparticles. (C)- (F) Photothermal effect and remaining time duration of the MCD in mouse joints. (G) - (I) Semiquantitative comparisons of inflammatory factors (TNF-α, IL-1β, and IL-8), therapy outcomes (three proteins associated with OA pathology and cartilage reconstruction), composite scores, movement distance, and velocity in OA joints among different treatment methods. Figures adapted with permission from Ref. 95. Copyright © 2019, American Chemical Society.

Zhao *et al.* designed another nanoprobe, MoS_2_@CS@Dex (MCD, chitosan (CS)-modified molybdenum disulfide (MoS_2_) loaded with anti-inflammatory disulfide (Dex)), which significantly increased the intra-articular drug release efficiency in OA joints with the satisfactory photothermal conversion efficiency of the drug carrier 95. In general reaction process shown in Figures 10(A-B), after injection of the nanoparticles and under the NIR infrared illumination, drugs loaded in the MCD are released in the inflamed areas and induce activated macrophage, which further results in reduction of inflammatory factors to realize the treatment goals. The photothermal properties and photoacoustic signals fluctuations shown in Figures 10(C-F) suggest that the MCD has a sound photothermal conversion ability and its long retaining period in OA joints helps to maintain an efficient drug release. Semiquantitative results shown in Figures 10(G-I) further confirm that the MCD, as an efficient photothermal contrast and drug carrier, can yield therapeutic outcomes superior to other treatment methods.

## Clinical application

**Figure 11:**
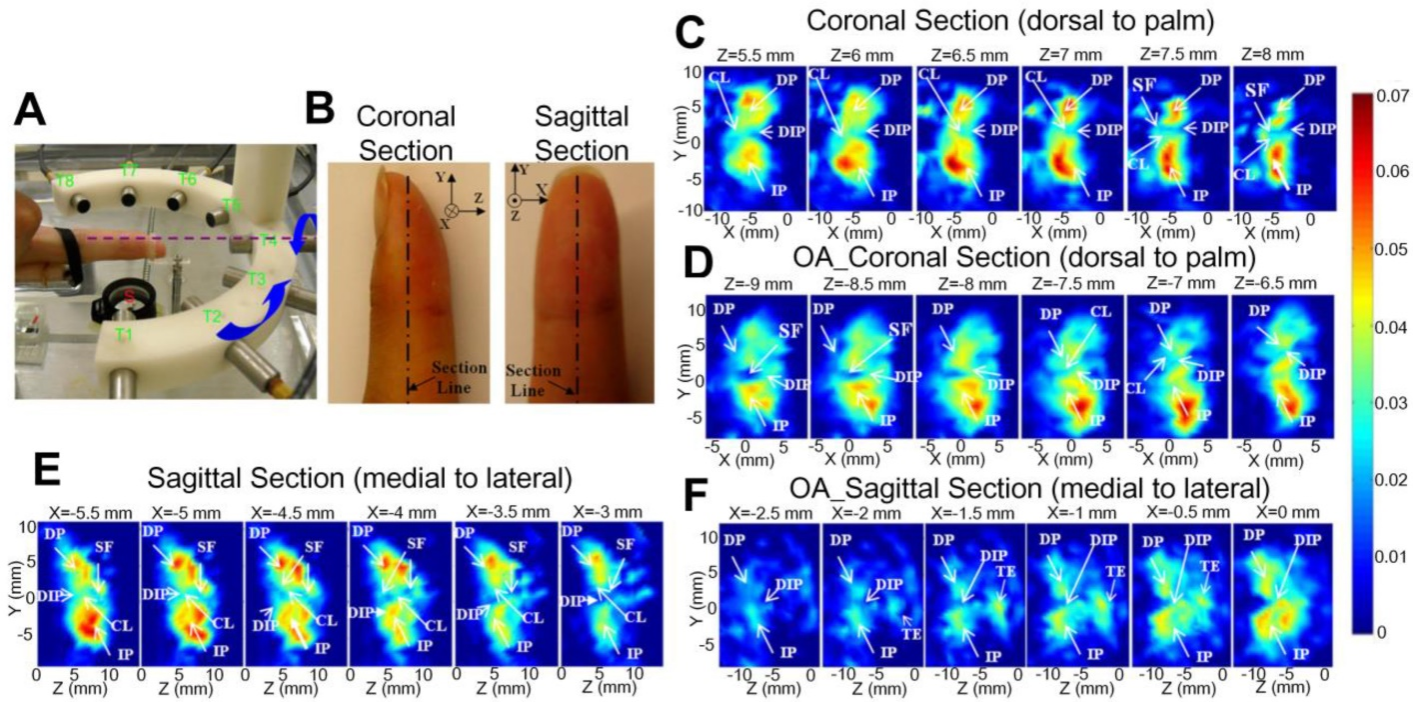
(A) Photograph of the PAT system. (B) Photograph of the finger joint through different sections. (C)-(D) PA recovered 3D absorption coefficient image of the corona section for normal and OA joints, respectively. (E)-(F) PA recovered 3D absorption coefficient image of the sagittal section for normal and OA joints, respectively. CL: cartilage; DIP: distal interphalangeal joint; DP: distal phalanx; IP: intermediate phalanx; SF: synovial fluid. Figures adapted with permission from Ref. 96. Copyright © 2011, American Association of Physicists in Medicine.

Sun *et al.* reported *in vivo* detection of OA in the finger joints via three-dimensional (3D) quantitative photoacoustic tomography (PAT) 96, 97, which uses a reconstruction algorithm to obtain absorption coefficient images of the joints. As illustrated in Figures 11(A-B), different sections, including both coronal and sagittal sections, of the fingers were imaged by the PAT. As seen, it might be hard to tell the structure difference between the healthy and OA joints from the PAT recovered 3D images, but they do show that the absorption coefficient has larger variations at different imaging depths for the OA finger joint cavity (Figures 11D and F), while for the healthy joint cavity the PA signals keep relatively constant at different depths (Figures 11C and E). It also can be found from the quantitative results that the space between joints also exhibits apparent differences for the OA and healthy joints. Similar observations have been reported from two-dimensional (2D) quantitative PA imaging with the same system proposed by this group, confirming the depth-dependent feature of tissue absorption coefficient for OA joints 98. They also founded that the functional parameters changes in OA joints compared with normal human fingers 81, as mentioned in Figure 5.

**Figure 12:**
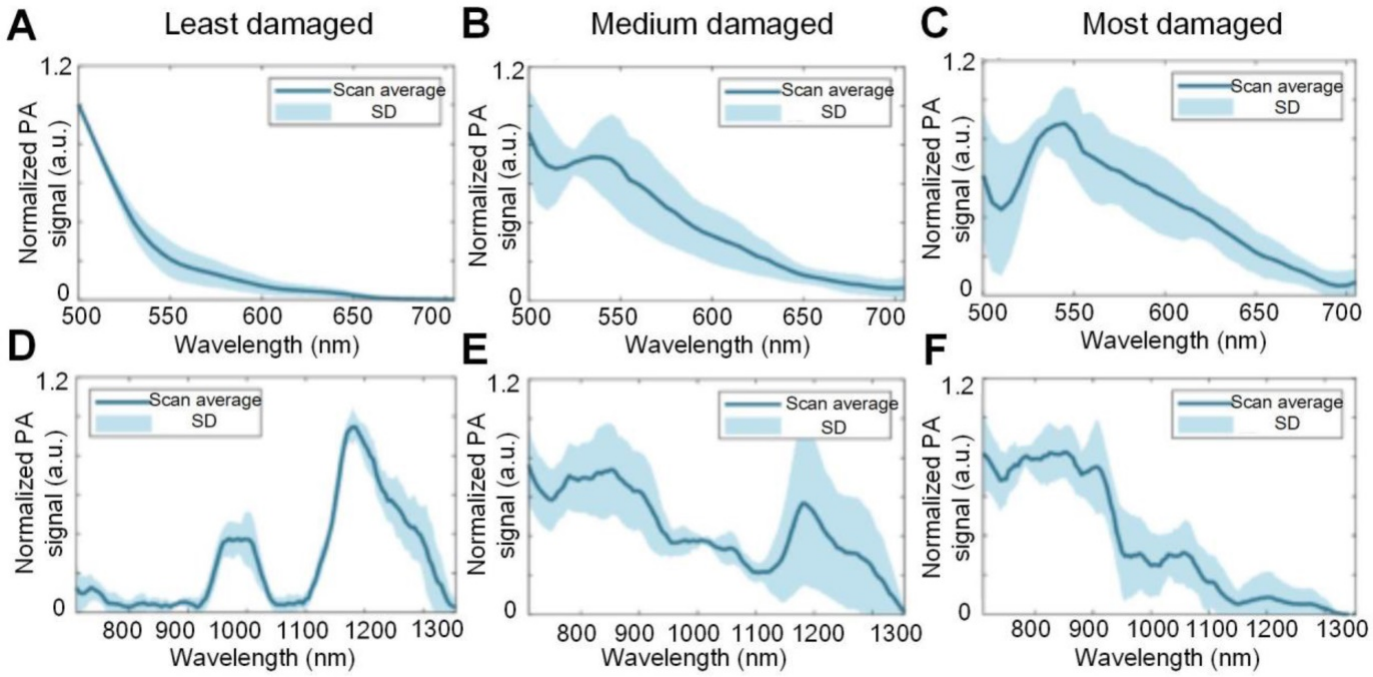
(A)-(C) PA spectra of different damaged cartilage (least damaged, medium damaged and most damaged) at 500 nm- 700 nm. (D)-(F) PA spectra of different damaged cartilage (least damaged, medium damaged and most damaged) at 710 nm- 1300 nm. Figures adapted with permission from Ref. 99. Copyright © 1969, Elsevier (1969).

The tissue absorption spectra, as reflected by the PA signal intensity changes as a function of optical wavelength, corresponding to cartilages of different degrees of damage has also been investigated. Wu *et al*. used the PA spectra changes to differentiate the damage degree of cartilages, and the results are shown in Figure 12 99. As seen, for the least damaged cartilage in the wavelength range from 500 nm to 1300 nm, there are two absorption peaks at 980 and 1185 nm, which are associated with the absorption peaks of water and collagen, respectively. But the spectrum profile changes with the degree of damage. It is hard to find the two above-mentioned absorption peaks in most damaged cartilage, suggesting a loss of balance between the water and collagen contents inside the damaged cartilage. Moreover, the elevated absorption around 550 nm for most damaged cartilage confirms the increased vascularization of OA as illustrated in Figures 2 and 4.

## Conclusion and perspectives

In this manuscript, we have narratively reviewed the applications of photothermal effects in the diagnosis and treatment of OA. Regarding diagnosis, photoacoustic imaging, a hybrid noninvasive imaging modality that integrates the advantages of optical (absorption) contrast with ultrasound detection, is considered. The working principle of PA imaging and current studies in cancers and brain activities are first described, which is followed by a summarization of its applications for diagnosis and status evaluation of OA joints from structural changes, functional parameters, as well as molecular information. With PA imaging, it is feasible to capture the structural changes in spongy bone and cartilage, allowing one to identify OA joints from normal joints. Functional parameters, including vascularity, oxygen saturation, hemoglobin concentration, water contents, acoustic velocities, and viscoelasticity, experience considerable variations even in the early stage of OA joints, which further causes changes of the optical spectra. Thus, it is possible to achieve early diagnosis and developmental stage evaluation of the OA disease with the assist of PA imaging. Moreover, PA can also be used to sense and then quantify the changes of molecular information. For example, the glycosaminoglycans concentration, an important indicator of the cartilage denegation degree, can be captured by PA with specifically designed nanoprobes. Collectively, with the capability of structural, functional, and molecular imaging, PA provides a promising tool for early-stage detection and timely treatment of OA.

Regarding the treatment of OA, PTT is reviewed in this work. As a relatively new therapeutic technology, PTT has seen wide applications in cancer treatment but only a few in OA joints treatment. But the noninvasive feature and confirmed enhanced therapeutic ability of PTT have made it distinctively potential for the treatment of OA joints treatment. Moreover, the therapeutic precision, efficiency, and recovery outcomes can be further enhanced with the help of molecule-targeted contrast agents. Lastly, the relevant clinical applications including analyzing changes in tissue absorption coefficient and absorption spectrum associated with human OA have been discussed.

That said, at current stage the clinical trials of PA and PTT in OA diagnosis or treatments are yet limited within a shallow penetration depth (e.g., finger) in human body, and the research models are mainly aiming at animals. Taking consideration of the trade-off between penetration depth and spatial resolution, how to achieve high resolution and deep penetration simultaneously so that PA imaging can be extended for clinics is an important challenge, which holds great significances in clinical diagnosis for OA and many other diseases.

There are two potential strategies to tackle the challenge. One is to explore if the diffusive light at depths in joints can be refocused so as to enhance the *in-situ* photon flux and hence the PA signal strength. Note that PA imaging has indeed reduced the influence of optical scattering by converting light, diffused or not diffused, into non-scattered ultrasonic waves due to optical absorption, which, however, only applies to the signal detection aspect. For the generation of signals, the amount of photons reaching the region of interest that can be absorbed and converted into heat and hence the effective penetration depth in PA are stilled throttled by the strong scattering in the tissue. Optical wavefront shaping can compensate for the scattering-induced phase distortion of light and generate optical focusing through or within a highly scattering medium 100, 101. Especially, when photoacoustic signal is used the guidestar for optimization 102, acoustical- or even optical-diffraction limited light focusing may be achieved at the focal position of the ultrasound transducer at depths in tissue, which can significantly enhance the signal strength and hence the penetration depth. The feasibility of such a concept of photoacoustically guided wavefront shaping for enhanced photoacoustic imaging has already been demonstrated in tissue-mimicking phantom and ex-vivo tissue 100, 103, 104, and it is currently under development towards in-vivo applications. Once it is mature, it will allow more reliable PA diagnosis of OA even at depths. The other possible solution is applying deep learning to achieve enhanced PA resolution in deeper tissue regions. Deep learning has seen wide applications in bioimaging including PA imaging in recent years due to its excellence in achieving super resolution imaging 105-107. For example, in PA imaging, deep learning assistance has enabled 100% decrease in background noise and up to 250% increase in signal strength 108. Moreover, the trained network has the capability to intelligently highlight the target and reduce the fuzzy information via big data learning. Currently, the state-of-art PACT has been successfully applied to human breast and brain trials *in vivo* 18, 109, albeit with limited resolution. In application, the neural network can be designed and trained based on co-registered low-resolution PACT and high-resolution PAM data acquired from OA joints in relatively shallow tissue regions, Then, the transformation capability of the network can be extended to deep tissue regions that only allows for PACT, producing high-resolution PA images of OA joints that cannot be physically obtained with existing PA implementations.

Apart from the penetration depth, the sensitivity of existing nanoprobes could be further improved to enhance the detection sensitivity and specificity. Current nanoprobes as the photoacoustic or photothermal contrasts mostly conjugate to the OA regime passively via permeability or microenvironment variations. These nanoparticles may also gather in the surrounding regions, reducing the contrast, precision, or the efficiency of photoacoustic diagnosis or photothermal-triggered treatment. If key molecules for early OA can be identified and active targeting probes can be synthesized, PA can potentially precisely locate the lesion of early OA, which can also considerably increase the treatment efficiency through photothermal-triggered therapy while minimizing the damage to surrounding normal tissues. Since PA and PTT share the same photothermal mechanism, it is feasible to integrate these two modules into one system that can achieve precise detection of early-stage OA joints with PA and timely treatment with PTT simultaneously, providing a promising theranostic platform for OA joints.

On the other side, the natural cavity structure of human joints provides another opportunity for PA imaging to bypass the technical challenges of tissue penetration depth to achieve high-resolution information down to the molecular level. The joint cavity is alike in hollow structure in the cardiovascular and gastrointestinal systems. In this regard, nanorobots can be further developed for the human joint based on photoacoustic endoscopy (PAE) for blood vessels [110]and the digestive tract 111. By doing so, we might be able to overcome the obstacles in the transmission of sound waves to achieve micron-level resolution at millimeter-level imaging depths. Moreover, the imaging nanorobots shall be designed for specific imaging objects and imaging ranges with the multimodal functional system. It would be better at finding the location of the lesion timely, providing the doctor with images of the lesion area, guiding the operation, monitoring, collecting specimens, and removing the location of the lesion inside osteoarthritic joint at its early stage of disease.

Although there have been quite a few attempts to deploy PA for OA imaging, the majority of these studies remain limited to proof-of-concept in the laboratory setting. Some nano-contrast agents for PA molecular imaging play a role in OA diagnosis, nevertheless, there are still many issues to be further addressed before clinical translation, such as their immunogenicity, toxicity, particle size, *etc.* Among 49 clinical trials related to PA technology in the ClinicalTrials.gov database, there are only two ongoing clinical trials for rheumatoid arthritis (one in China 112, another one in the United States 113) but none for OA. Although there is still a long way ahead, PA holds a good promise for clinical translation. It prompts the needs for joint efforts among engineers, clinicians and their industrial partners along this direction.

In conclusion, PA imaging technology can not only perform specific structure imaging of tissues with high resolution and high contrast, identify specific components in tissues, but also can combine molecular nanorobots for targeted imaging, showing huge potential clinical market value. It has great economic value and social significance, promoting the further clinical transformation and industrialization of PA imaging technology in OA theranostic. We hope this mini review can provide a better understanding of the diagnosis and treatment of OA with exciting innovations based on tissue photothermal effects. It may also inspire more explorations in the field towards earlier and better theranostic of OA.

## Figures and Tables

**Table 1:** Summary of current imaging modalities' capacities for OA diagnosis

Imaging modality	Capacities
X-ray	Evaluation of osteophyte formation, joint space narrowing (JNS), and subchondral sclerosis or subchondral cysts. 4-6
PET	Provide metabolism information in articular and periarticular tissues. 5
MRI	Offer superb soft tissue contrast, which is used in assessment of cartilage lesions. 4-6
OCT	Generate cross-sectional images of articular cartilage and provide quantitative information about the disease state of articular cartilage; sensitive to collagen structural changes. 4-6
US	Identification of tibiofemoral osteophytes, medial meniscal extrusion, and medial femoral cartilage morphological degeneration 4-6

## Abbreviations

OA: osteoarthritis
X-ray: radiography
PET: positron emission tomography
MRI: magnetic resonance imaging
OCT: optical coherence tomography
US: ultrasound
JSW: joint space width
JNS: joint space narrowing
SPECT: single-photon emission computed tomography
PA: photoacoustic
PAM: photoacoustic microscopy
PACT: photoacoustic computed tomography
SNR: signal-to-noise ratio
PTT: photothermal therapy
MMPs: matrix metalloproteinases
NO: nitric oxide
ROS: reactive oxygen species
TNF-α: tumour necrosis factor-α
IL-1β: interleukin-1β
PD: power doppler
DMM: medial meniscus
HbO_2_: oxyhemoglobin
Hb: deoxyhemoglobin
DIP: distal interphalangeal
SERS: surface-enhanced Raman scattering
LIPA: laser-induced PA
GAGs: glycosaminoglycan
PLL-MNPs: poly-_L_-lysine-encapsulated melanin nanoparticles
MNP: melanin nanoparticles
HA: hyaluronic acid
NHsPP: NO-Hb@siRNA@PLGA-PEG
MCD: MoS_2_@CS@Dex
CS: chitosan
MoS_2_: molybdenum disulfide
Dex: disulfide
3D: three-dimensional
PAT: photoacoustic tomography
2D: two-dimensional
PAE: photoacoustic endoscopy
